# Bonding Impairment as an Explanatory Link between Unplanned Pregnancy and Maternal Infant Abuse: Results of a Causal Mediation Analysis

**DOI:** 10.31662/jmaj.2024-0277

**Published:** 2025-09-26

**Authors:** Emily Ivanich, Nobutoshi Nawa, Aya Goto, Takeo Fujiwara, Pamela J. Surkan

**Affiliations:** 1 Centers for American Indian and Alaska Native Health, Colorado School of Public Health, Aurora, Colorado, United States of America; 2 Department of Global Health Promotion, Tokyo Medical and Dental University (TMDU), Tokyo, Japan; 3 Center for Integrated Sciences and Humanities, Fukushima Medical University, Fukushima, Japan; 4 Department of International Health, Johns Hopkins Bloomberg School of Public Health, Baltimore, Maryland, United States of America

**Keywords:** pregnancy, maternal bonding, unplanned pregnancy, public health, violence

## Abstract

**Introduction:**

Unplanned pregnancy is associated with maternal-infant abuse. We investigated the association between unplanned pregnancy and maternal abusive behaviors and whether this relationship is mediated by bonding impairment.

**Methods:**

We analyzed data from a sample of mothers with 3-4-month-old infants in Japan (n = 5,706). Multivariable logistic regression models were used to assess the associations among unplanned pregnancy, bonding impairment, and maternal-infant abuse. Causal mediation analysis was conducted to estimate the mediating effect of bonding impairment on the relationship between unplanned pregnancy and maternal-infant abuse.

**Results:**

Among mothers who engaged in infant abuse, 24.8% reported an unplanned pregnancy, compared to 17.7% among those who did not. Unplanned pregnancy was significantly associated with an increased risk of maternal-infant abuse in a multivariable analysis (adjusted odds ratio [OR] = 1.36; 95% confidence interval [CI], 1.02-1.80). After adjusting for bonding impairment as a mediator, the association was attenuated (OR = 1.28; 95% CI, 0.96-1.70). Causal mediation analysis revealed a natural direct effect of unplanned pregnancy on maternal-infant abuse (OR = 1.28; 95% CI, 0.96-1.70), as well as a natural indirect effect through bonding impairment as a mediator (OR = 1.06; 95% CI, 1.03-1.09). Bonding impairment accounted for 20.1% of the association between unplanned pregnancy and maternal-infant abuse.

**Conclusions:**

Bonding impairment partially mediates the relationship between unplanned pregnancy and maternal-infant abuse.

## Introduction

Victims of child abuse often experience lasting harm into their adulthood, with collateral effects that may extend to future generations ^(1)^. The consequences of abuse negatively affects victims’ physical, psychological, and behavioral health ^(2), (3)^. These outcomes include an increased risk of diabetes, malnutrition, depression, anxiety, delinquency, substance use, and the intergenerational perpetuation of child abuse ^(2), (3)^.

Child abuse also imposes a significant burden on society. For instance, the lifetime medical costs of child maltreatment in Japan have been estimated at ¥333 billion (US dollar [USD] 3.1 billion) annually ^(4)^. When indirect costs―such as lost productivity―are included, in addition to the medical expenses of child maltreatment, one 2016 study in Japan estimated the total cost to be USD 50 billion ^(5)^. These financial burdens appear to persist across the life span; among Japanese adults aged 65-75, those with a history of childhood maltreatment incurred an average of ¥136,456 (USD 1,255) more in medical expenses compared to those without such a history ^(4)^. A study by Arai et al. ^(6)^ suggests that these financial disadvantages may increase the risk of maternal maltreatment toward children.

Among various factors contributing to child abuse, Japanese mothers with unplanned pregnancies are more than twice as likely to engage in maternal-infant abuse compared to mothers with planned pregnancies ^(7)^. Although abortion is legal until up to 22 weeks of gestation, permission of the spouse is needed for married women (Ministry of Health, Labour and Welfare, 1996), which may contribute to the prevalence of unwanted pregnancies, if the spouse does not allow for an abortion ^(8)^. Unplanned pregnancies are associated with numerous adverse outcomes for both children and mothers in Japan ^(9)^. In particular, impaired maternal-infant bonding may be exacerbated by unplanned pregnancies or by mothers’ negative attitudes toward pregnancy ^(10)^. Kokubu et al. ^(10)^ (2012) identified a positive association between negative attitudes toward pregnancy and bonding impairment in Japanese mothers. Similarly, Kuroda et al. ^(11)^ found that bonding during and after pregnancy was linked to maternal mental health. They suggested that promoting positive attitudes toward pregnancy could help prevent postpartum depression, which is associated with parenting behaviors ^(11)^. Collectively, these findings led us to hypothesize that impaired maternal-infant bonding may serve as a pathway through which unplanned pregnancy could lead to maternal-infant abuse.

Understanding factors that can mediate maternal-infant abuse is paramount to preventing maternal-infant abuse among women with unplanned pregnancies. To our knowledge, no existing studies have examined bonding impairment as a mediator between unplanned pregnancy and maternal-infant abuse. Therefore, the primary aim of this study is to investigate the indirect effects of maternal-infant bonding in mediating the relationship between unplanned pregnancy and maternal-infant abuse. A secondary aim is to examine the direct effects of unplanned pregnancy and bonding impairment on maternal-infant abuse.

## Materials and Methods

### Study design

Data collection was conducted in Aichi Prefecture, Japan, between October and November 2012. Of the 54 municipalities in the prefecture, 45 (covering approximately 80% of Aichi Prefecture) agreed to participate. Mothers who had been registered in a program for their child’s 3-4-month health check-up during that period were invited to participate. This health program included 97.7% of eligible women in the participating 45 municipalities (n = 9,707). Of those invited, 6,590 caregivers responded (68% response rate). After excluding cases with missing data and non-meaningful responses, the final sample size was 5,706.

In 34 municipalities, questionnaires were mailed directly to individual participants, and completed questionnaires were collected at the check-up sites. In the remaining 11 municipalities, questionnaires were distributed at the check-up sites and later mailed back by participants to the health center.

The Ethics Committee of the National Center for Child Health and Development in Tokyo, Japan, approved this study (reference number 611). All participants provided informed consent prior to participation.

### Measures

#### Dependent variable

Maternal-infant abuse was assessed through caregiver responses to questions about shaking or smothering their baby in the past month. For shaking, we asked: “When your child is crying and making a fuss, how many times have you shaken your child violently?” For smothering, we asked: “When your child is crying and making a fuss, how many times have you used your hand or a cushion, etc., to cover your child’s mouth?” Possible responses to both questions included: “0 times,” “1-2 times,” “3-5 times,” “6-10 times,” and “11 or more times.” Due to data skewness, we dichotomized responses into “no” ( for participants who answered “0 times) and “yes” (for participants who gave any other response). Additionally, we combined the shaking and smothering variables into one variable indicating any “maternal-infant abuse.” Participants who answered “yes” to either the shaking or smothering variables (or both) were classified as “yes,” while those who answered “no” to both were classified as “no” ^(12)^.

#### Independent variable

We used a Japanese screening tool originally designed to detect risk of child abuse to assess mothers’ feelings and intentions toward their pregnancy ^(11)^. To evaluate pregnancy intention, we asked: “How did you feel when you found out about this pregnancy?” Responses included in the analysis were “Happy,” and “Happy, as it was unexpected.” We excluded responses such as “Confused, as it was unexpected,” “Troubled,” “Nothing in Particular,” and “Other” because they did not clearly indicate whether the pregnancy was planned or unplanned and reflected mixed emotional reactions that were difficult to categorize. The included response categories were dichotomized into intended (“Happy”) and unplanned pregnancy that included “Happy, as it was unexpected.” Women who are happy about a pregnancy but did not plan for it may still feel unsupported or unprepared―financially, emotionally, due to an unsupportive partner, or for other reasons ^(13), (14), (15)^. This lack of preparation may affect their ability to bond with their child. We are interested in exploring how the timing and preparedness of a pregnancy influence mother-child bonding.

#### Mediator variable

We assessed bonding impairment using the Japanese version of Kumar’s Mother-Infant Bonding Questionnaire ^(16), (17)^. Ten traits related to mother-infant bonding were evaluated by asking mothers questions about their 3-4-month-old child. Participants responded using a four-point Likert scale: “I really think so” (assigned 0), “I sort of think so” (assigned 1), “I don’t really think so” (assigned 2), and “I don’t think so at all” (assigned 3). Selected items were reverse-coded so that higher scores indicated greater bonding impairment. A total score above 4 was considered indicative of bonding impairment ^(18), (19)^.

#### Covariates

The covariates assessed were primipara, whether or not the mother returned to her hometown for delivery (a common practice in Japan known as *satogaeri bunben*), household composition (whether the infant lived with one or both parents or non-parental relatives), maternal age, self-perceived financial stability (whether they viewed their finances as either good or bad), and marital status. Postpartum depressive symptoms were measured using the Edinburgh Postnatal Depression Scale, a 10-item questionnaire with four response options per item ^(20)^, yielding a total score from 0 to 30. A score of ≥9 was used as the cutoff for probable postpartum depression ^(21)^.

### Data analysis

We calculated descriptive statistics for all variables. Logistic regression analyses were conducted to estimate associations between unplanned pregnancy and maternal-infant abuse, and between bonding impairment and maternal-infant abuse. In addition to unadjusted models for both predictors, we built adjusted models controlling for postpartum depressive symptoms, primipara, returning to maternal hometown for delivery, household composition, maternal age, financial stability, and marital status. Unplanned pregnancy and bonding impairment were entered separately in the crude and adjusted models. We also adjusted for all covariates and included unplanned pregnancy and bonding impairment in the model simultaneously. Causal mediation analysis was conducted using the paramed command in Stata SE statistical package, version 14 (StataCorp LP, College Station, TX, USA) ^(22), (23)^. Causal mediation analysis examined whether bonding impairment mediated the relationship between an independent variable and a dependent variable, using a counterfactual framework ^(22), (23)^. The total effect of unplanned pregnancy on maternal-infant abuse was divided into natural indirect effects (NIE), mediated through bonding impairment, and natural direct effects (NDE), not mediated through bonding impairment. All covariates were adjusted for in the mediation analysis. The proportion mediated was calculated using equations for models with binary mediators and binary outcomes ^(22)^.

## Results

Table 1 presents descriptive statistics. After excluding missing data, the final sample included 5,706 participants. Specifically, 103 participants were excluded due to missing data on maternal-infant abuse; 71 for missing data on unplanned pregnancy; and 454 for selecting non-meaningful responses to the unplanned pregnancy variable. Additionally, 62 participants were excluded due to missing data on bonding impairment variable; 56 for missing probable postpartum depression data; 71 for missing in the primipara variable; and 75 for missing data on the *satogaeri bunben* variable. No exclusions were made based on the household members variable. Further, 35 participants were excluded due to missing maternal age variable, 311 for missing financial stability variable, and 46 for missing marital status. Among the remaining 5,706 participants, approximately 18.0% reported unplanned pregnancies, and 421 (7.4%) mothers were identified as having impaired bonding. Likewise, 463 (8.1%) participants screened positive for probable postpartum depression. Half the sample were first-time mothers (50.5%), and nearly half gave birth in their hometowns (42.6%). Regarding adult household composition, the majority of respondents (84.9%) reported that the baby lived with both parents, while (4.5%) reported living with a single parent. The remaining 10.6% reported that the child lived in a home with some other household make-up such as households with other relatives such as grandparents, aunts, and uncles.

**Table 1.** Baseline Characteristics of Study Participants (n = 5,706).

	Total	Combined shaking and smothering		
		One or both	Neither	p-Value
Characteristics	(n = 5,706)	(n = 290)	(n = 5416)	
	n (%)	n (%)	n (%)	
Unplanned pregnancy	1,028 (18.0%)	72 (24.8%)	956 (17.7%)	0.002
Bonding impairment	421 (7.4%)	61 (21.0%)	360 (6.6%)	<0.001
Probable postpartum depression	463 (8.1%)	54 (18.6%)	409 (7.6%)	<0.001
Primiparous	2,881 (50.5%)	194 (66.9%)	2,687 (49.6%)	<0.001
Returned to hometown for delivery^a^	2,429 (42.6%)	141 (48.6%)	2,288 (42.2%)	0.032
Household members				
Both Parents	4,844 (84.9%)	216 (74.4%)	4,628 (85.5%)	<0.001
Single Parent	257 (4.5%)	27 (9.3%)	230 (4.2%)	
Other	605 (10.6%)	47 (16.2%)	558 (10.3%)	
Maternal age				
≤24	412 (7.2%)	46 (15.9%)	366 (6.8%)	<0.001
25-34	3,762 (65.9%)	194 (66.9%)	3,568 (65.9%)	
≥35	1,532 (26.9%)	50 (17.2%)	1,482 (27.4%)	
Financially Stable	5,098 (89.3%)	247 (85.2%)	4,851 (89.6%)	0.018
Unmarried	65 (1.1%)	8 (2.8%)	57 (1.1%)	0.008

p-Values were calculated using a χ^2^ test. ^a^Refers to *satogaeri bunben*, is a traditional Japanese practice in which a pregnant woman returns to her parents’ home before giving birth and stays there for several weeks or months postpartum. This custom is deeply rooted in Japanese culture and reflects the importance of family support during childbirth and early motherhood.

Most mothers were between 25 and 34 years of age (65.9%), and approximately one-quarter were older than 35 (26.9%). Only a small proportion were 24 years old or younger. Nearly all participants self-reported financial stability (89.3%), and a small minority (1.1%) reported being unmarried at the time of data collection.

In Table 1 we compare the characteristics of mothers who engaged in maternal-infant abuse behaviors (i.e., either shaking and/or smothering) versus those who did not. Among those who engaged in abuse, 24.8% reported unplanned pregnancies, compared to 17.7% among those who did not (p = 0.002). Additionally, 21.0% of mothers who engaged in abuse were bonding impaired, compared to just 6.6% among those who were not abusive (p < 0.001). Probable postpartum depression was present in 18.6% of abuser caregivers, versus 7.6% in their non-abusive counterparts (p < 0.001).

**Figure 1.**
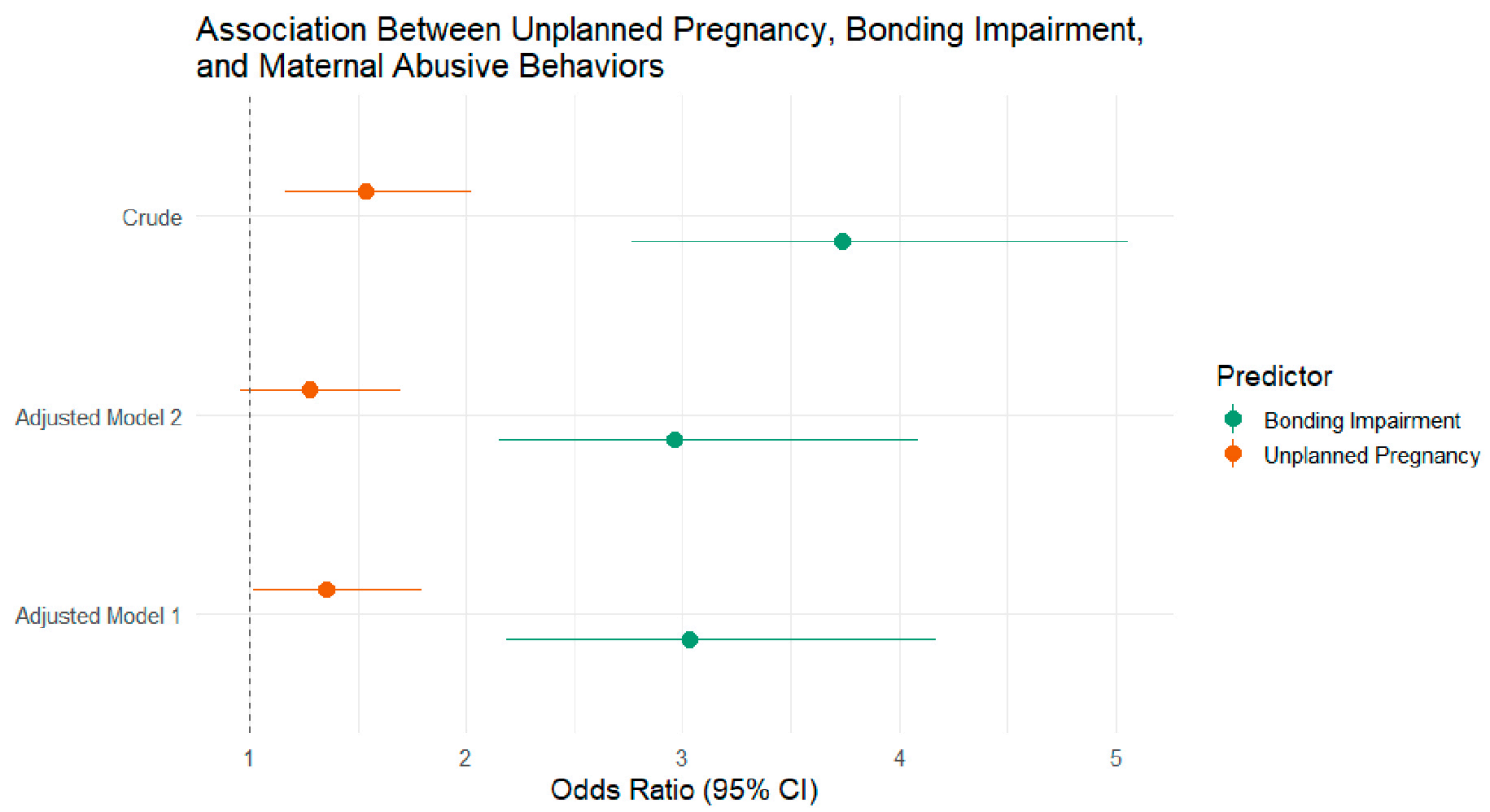
Association between unplanned pregnancy and maternal abusive behaviors. CI: confidence interval. *Adjusted Model 1*: Unplanned pregnancy and bonding were entered into the model separately. The model was adjusted for probable postpartum depression, primiparous, hometown delivery (satogaeri bunben), household structure, age of mother, marital status, and financial stability. *Adjusted Model 2*: Unplanned pregnancy and bonding impairment were added into the model simultaneously. The model was adjusted for covariates in Adjusted Model 1.

Table 2 displays the associations between unplanned pregnancy and bonding impairment with maternal-infant abuse. Unplanned pregnancy was significantly associated with an increased risk of maternal-infant abuse both in crude (odds ratio [OR] = 1.54; 95% confidence interval [CI], 1.17-2.03) and multivariable-adjusted models (OR = 1.36; 95% CI, 1.02-1.80), which controlled for probable postpartum depression, primipara, delivery in the maternal hometown, household members, maternal age, financial stability, and marital status However, the association lost statistical significance after adjusting for bonding impairment and all covariates (OR = 1.28; 95% CI, 0.96-1.70). In contrast, bonding impairment remained statistically significant across models: independently (OR = 3.74; 95% CI, 2.77-5.06), adjusted for covariates without unplanned pregnancy (OR = 3.03; 95% CI, 2.19-4.17), and adjusted for both unplanned pregnancy and all covariates (OR = 2.96; 95% CI, 2.15-4.09) as seen in Figure 1.

**Table 2.** Unplanned Pregnancy and Bonding Impairment in Relation to Maternal Abusive Behaviors (n = 5,706).

Characteristics	Crude	Adjusted model 1^a^	Adjusted model 2^b^
	OR (95%CI)	OR (95%CI)	OR (95%CI)
Unplanned pregnancy	1.54 (1.17-2.03)^c^	1.36 (1.02-1.80)^c^	1.28 (0.96-1.70)
Bonding impairment	3.74 (2.77-5.06)^c^	3.03 (2.19-4.17)^c^	2.96 (2.15-4.09)^c^

CI: confidence interval; OR: odds ratio. ^a^Unplanned pregnancy and bonding were entered into the model separately. The model was adjusted for probable postpartum depression, primiparous, hometown delivery (*satogaeri bunben*), household structure, age of mother, marital status, and financial stability. ^b^Unplanned pregnancy and bonding impairment were added into the model simultaneously. The model was adjusted for covariates in adjusted model 1. ^c^p < 0.05.

**Figure 2.**
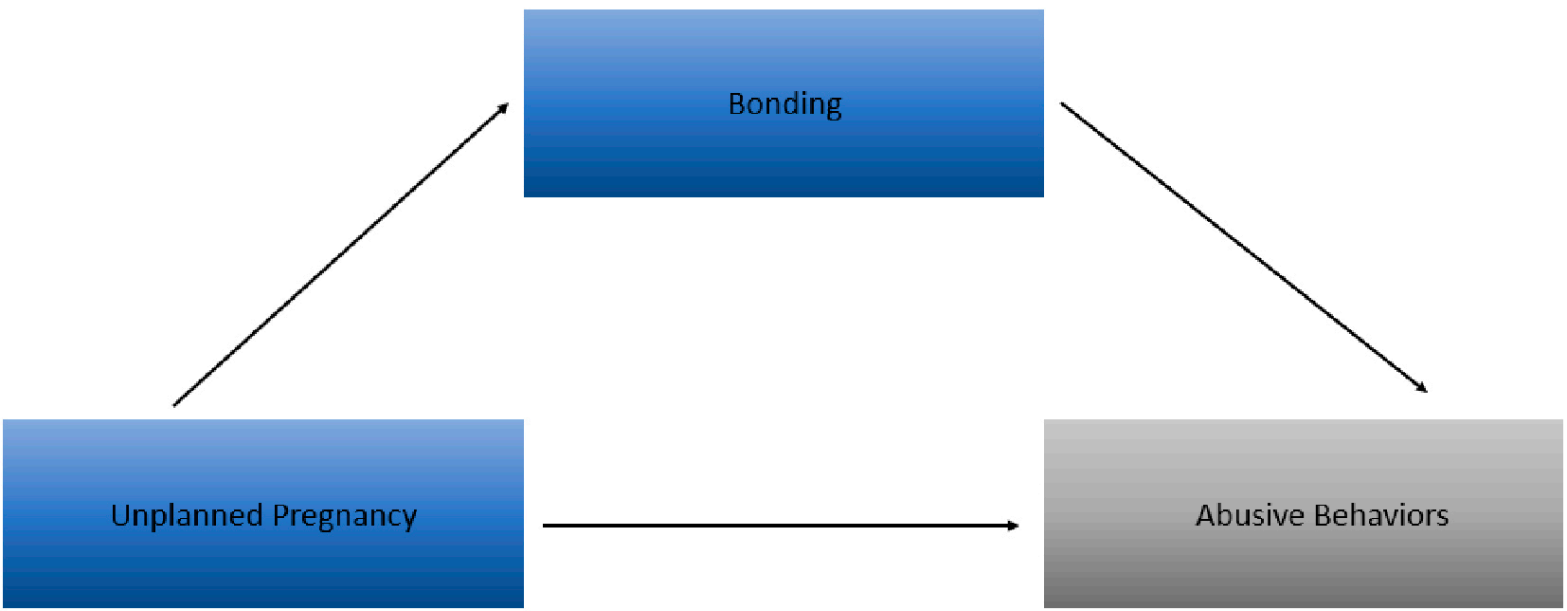
Mediation analysis results: effects of unplanned pregnancy on maternal abusive behaviors This diagram illustrates the mediation model examined in our study. The arrow from *Unplanned Pregnancy* to *Abusive Behaviors* represents the direct effect, while the pathway through *Bonding* represents the indirect effect. We found that bonding impairment was a potential mediator, approaching statistical significance, in the relationship between unplanned pregnancy and maternal-infant abuse. This suggests that strengthening maternal-infant bonding may help mitigate the risk of abuse.

Table 3 presents the results of causal mediation analysis examining the natural direct and indirect effects of unplanned pregnancy on maternal-infant abuse, with bonding impairment as a mediator. The NDE approached statistical significance (OR = 1.28; 95% CI, 0.96-1.70), while the NIE was statistically significant (OR = 1.06; 96% CI, 1.03-1.09). Bonding impairment accounted for 20.1% of the association between unplanned pregnancy and maternal-infant abuse. Figure 2 illustrates the mediation model of unplanned pregnancy on abusive behaviors with bonding as a mediator.

**Table 3.** Result of Mediation Analyses: Natural Direct and Indirect Effects of Unplanned Pregnancy on Maternal Abusive Behaviors with Bonding Impairment as a Mediator (n = 5,706).

Characteristics	Natural direct effect	Natural indirect effect^a^
	OR (95% CI)	OR (95% CI)
Unplanned pregnancy	1.28 (0.96-1.70)	1.06 (1.03-1.09)^b^

Adjusted covariates were probable postpartum depression, first time mothers, hometown delivery (*satogaeri bunben*), household structure, age of mother, marital status, and financial stability. CI: confidence interval; OR: odds ratio. ^a^Mediated through bonding impairment ^b^p < 0.05.

## Discussion

Our study demonstrated both unplanned pregnancy and bonding impairment were associated with an increased likelihood of maternal-infant abuse. Importantly, our findings extend the existing literature by suggesting that mother-infant bonding is a key factor in explaining the link between unplanned pregnancy and maternal-infant abuse. This is particularly noteworthy, as it implies that strengthening maternal-infant bonding in mothers with unplanned pregnancies―which accounted for nearly one in five pregnancies in our sample―may help reduce the risk of abuse.

After accounting for bonding impairment, the odds of maternal-infant abuse associated with unplanned pregnancy decreased from 1.36 to 1.28 and were no longer statistically significant. Our mediation analysis revealed that bonding impairment explained 20.1% of the relationship between unplanned pregnancy and maternal-infant abuse. These results underscore the importance of screening for unplanned pregnancy―especially in terms of timing―during the antenatal period. Early identification allows healthcare providers to deliver timely interventions that promote maternal-fetal bonding and, ultimately, healthier postnatal mother-infant relationships. To our knowledge, this is the first study to examine the mediating role of bonding impairment in the relationship between unplanned pregnancy and maternal-infant abuse. Other researchers in Japan have explored the link between intimate partner violence (IPV) and both mother-infant bonding ^(24)^ and child abuse ^(25)^. Given that IPV can lead to unplanned pregnancy ^(26)^, the relationship between unplanned pregnancy and maternal-infant abuse may mirror the association between IPV and child abuse. In a previous study using the same Aichi Prefecture data, our group found that maternal depressive symptoms mediated the relationship between IPV and bonding ^(24)^. Additional research is needed to better understand the reciprocal dynamics among these factors, including the role of depression―which we controlled for in our models―within the context of unplanned pregnancy and abuse.

We found a significant association between unplanned pregnancy and child abuse, consistent with prior studies in Japan and other countries ^(7)^. A study conducted in Nagoya City, Japan, found that women who had not intended to become pregnant were more likely to engage in shaking or smothering behaviors, particularly among mothers younger than 25 ^(7)^. Some have theorized that maltreatment may be a consequence of parental rejection―that is, when an unwanted pregnancy persists as an unwanted child ^(27)^. In the US, mothers who had considered abortion were over twice as likely to use extreme psychological aggression toward their children ^(27)^.

In this study, we limited the definition of unplanned pregnancy to participants who responded “Happy, as it was unexpected,” excluding those who selected “Confused, as it was unexpected” or other ambiguous choices. This decision improved the clarity of our exposure definition by focusing on clearly unplanned but emotionally positive pregnancies. While this enhanced conceptual consistency, it reduced our analytic sample size and may limit generalizability. Participants who felt confused about their pregnancy could have distinct psychosocial profiles not reflected in our findings. Future studies should explore more nuanced classifications of pregnancy intention and emotional response to better understand their implications for bonding and parenting behaviors.

Mothers with bonding impairment had a threefold increased risk of maternal-infant abuse. Bonding impairment has previously been identified as a predictor of various negative outcomes, including child abuse ^(28)^. Because bonding impairment can increase the risk of maternal-infant abuse, it may also adversely affect child development ^(29)^. According to Bowlby’s attachment theory, infants have an innate (not learned) drive to bond with caregivers ^(30)^. When this instinct is unmet, children may feel less secure in exploring their environment. Sidebotham and Heron used a proxy measure of parent-infant bonding by counting the number of positive attributes parents endorsed (from seven options) for their four-week-old infants ^(28)^. Parents who selected fewer positive traits were more likely to have children placed under child protection services by age six ^(28)^. Notably, bonding during pregnancy has also been shown to improve maternal mental health and reduce the risk of child abuse ^(28), (29)^. These findings emphasize the protective role of maternal bonding in preventing child abuse.

Our study has both strengths and limitations. First, our reliance on self-reported data may have affected accuracy, particularly for sensitive topics like maternal-infant abuse, mother-infant bonding, and the desire to become pregnant. We collected our data cross-sectionally instead of prospectively. Women with unplanned pregnancies tend to have more positive views of their children over time―especially if strong relationships are formed―and may differentially report unplanned pregnancies as having been intended ^(31)^. Such misreporting would likely weaken the observed associations. We focused on women who reported being happy about their pregnancy and compared the differences related to their pregnancy planning status. This restriction limits the generalizability of the results. Nonetheless our results highlight the importance of utilizing interventions to increase maternal bonding with their babies to prevent child abuse, even among women with initially positive emotions about unplanned pregnancies. Future studies should use prospective longitudinal designs to assess pregnancy intention before delivery, allowing for clearer classification. One strength of our study is its high response rate and large sample size. We recruited 9,707 women in Aichi Prefecture, and 68% participated. As our study was conducted in Japan, additional research is needed to determine whether these findings generalize to other populations.

Our findings contribute to the improved understanding of factors that increase and mediate maternal-infant abuse. Specifically, we found that mother-infant bonding impairment partially mediates maternal-infant abuse among Japanese mothers with unintended pregnancies. Researchers have revealed that interventions can successfully improve maternal bonding ^(32)^. For example, a study in Japan found that increased hospital and home visits enhanced mother-infant interactions and supported infant mental health in preterm infants ^(33)^. Based on our findings, social programs, hospitals, birthing centers, and other institutions that work with new mothers should focus on improving mother-infant bonding, particularly among mothers with unplanned pregnancies. Given that unplanned pregnancy affected 18% of our sample―and rates as high as 40% have been reported in Japan ^(34)^―such interventions could benefit a substantial proportion of the population. If a mother with an unplanned pregnancy could receive support to help her better bond with her child, society could reduce maternal-infant abuse.

## Article Information

### Conflicts of Interest

Pamela J. Surkan holds a paid academic appointment at the Institute of Science Tokyo. This arrangement has been reviewed and approved by Johns Hopkins University in accordance with its conflict of interest policies.

### Author Contributions

Conception and design: Emily Ivanich, Pamela J. Surkan, and Takeo Fujiwara. Statistical analysis and manuscript drafting: Emily Ivanich and Nobutoshi Nawa. Critical review and supervision: Pamela J. Surkan, Nobutoshi Nawa, Aya Goto, and Takeo Fujiwara. Data collection: Takeo Fujiwara. All authors read and approved the final manuscript.

### Approval by Institutional Review Board (IRB)

The Ethics Committee of the National Center for Child Health and Development in Tokyo, Japan, approved this study (reference number: 611). All participants provided written informed consent prior to their participation.

### Disclaimer

Aya Goto is one of the Editors of JMA Journal and on the journal’s Editorial Staff. She was not involved in the editorial evaluation or decision to accept this article for publication at all.
